# Virtual Standardized Patients for Improving Clinical Thinking Ability Training in Residents: Randomized Controlled Trial

**DOI:** 10.2196/73196

**Published:** 2025-12-08

**Authors:** Liyuan Xu, Qinrong Xu, Chunya Liu, Baozhen Chen, Chunxia Wang

**Affiliations:** 1 Department of Endocrinology, Wenzhou Medical University Affiliated Quzhou Hospital, Quzhou People's Hospital, No. 100 Minjiang Avenue, Quzhou City, Quzhou, Zhejiang Province, 324002, China, 8618857008862

**Keywords:** virtual standardized patients, clinical competence, clinical thinking ability, medical education, medical students

## Abstract

**Background:**

Clinical internal medicine practice training traditionally relies on case-based teaching. This approach limits the development of students' clinical thinking skills. It also places significant pressure on instructors. Virtual standardized patients (VSPs) could offer an alternative solution. However, evidence on their feasibility and effectiveness remains limited.

**Objective:**

This study aims to use the “VSPs in general practice” interactive diagnostic and teaching system, which uses VSPs to provide 3D virtual simulated patients and mimic virtual clinical scenarios. Medical students are trained through system-preset cases. This study aims to establish the clinical application of VSPs through a “VSPs in general practice” system and compare its effectiveness with traditional teaching in improving students’ clinical thinking ability.

**Methods:**

A randomized controlled trial was conducted from October 20, 2022, to October 20, 2024. A total of 60 medical students interning at Quzhou People’s Hospital were enrolled and divided into 2 groups: the experimental group receiving VSP training (30/60, 50%) and the control group receiving traditional academic training (30/60, 50%). The teaching effectiveness was evaluated using basic knowledge assessments and virtual system scoring. After completing the course, students were surveyed with a questionnaire to assess their satisfaction with the course.

**Results:**

All enrolled medical students completed the study. In the evaluation of training effectiveness, the experimental group showed significantly greater improvement in theoretical scores compared to the control group (mean 17.07, SD 4.24 vs mean 10.67, SD 4.91; *F*_1, 59_=29.20; Cohen *d*=1.15; 95% CI 12.43-15.31; *P*<.001); the total score improvement in the virtual clinical thinking training system test was also significantly better in the experimental group than in the control group (mean 42.60, SD 9.56 vs mean 31.63, SD 7.24; *F*_1, 59_=25.10; Cohen *d*=1.09; 95% CI 34.51-39.72; *P*<.001). Specifically, improvements in consultation skills (mean 8.76, SD 1.67 vs mean 7.66, SD 2.08; *F*_1, 59_=31.09; Cohen *d*=0.55; 95% CI 7.70-8.70; *P*<.001), overall objective improvement (mean 11.97, SD 2.77 vs mean 8.15, SD 2.62; *F*_1, 59_=30.08; Cohen *d*=1.16; 95% CI 9.21-10.91; *P*<.001), initial diagnostic ability (mean 8.74, SD 1.67 vs mean 7.66, SD 2.08; *F*_1, 59_=4.91; Cohen *d*=0.55, 95% CI 7.70-8.70; *P*=.03), and ability to provide patient treatment (mean 7.23, SD 2.41 vs mean 5.72, SD 2.19; *F*_1, 59_=6.42; Cohen *d*=0.63; 95% CI 5.85-7.01; *P*=.01) were significantly higher in the experimental group than in the control group. The questionnaire results indicated that 90% (27/30) of the students who participated in the VSPs’ training believed it could enhance their clinical thinking abilities.

**Conclusions:**

VSPs reinforce the foundational knowledge of internal medicine among medical students and enhance their clinical thinking abilities, as well as improve their capacity for independent work. The VSP system is feasible, practical, and cost-effective, making it worthy of further promotion in clinical education.

## Introduction

Against the backdrop of the continuous expansion of higher medical education, clinical skills training is facing multiple developmental challenges. On one hand, medical schools are under real pressure to address the shortage of millions of health care professionals, urgently needing to scale up the training of high-quality clinical physicians [1]. On the other hand, they are constrained by the relative scarcity of high-quality teaching resources, leading to significant regional disparities in the quality of medical talent cultivation [2]. Clinical reasoning ability, as the core competency for medical students transitioning into qualified clinical physicians, essentially requires the integration of multidimensional clinical information such as patient history collection, physical sign identification, laboratory data interpretation, and imaging analysis [3]. However, the current clinical thinking training system still has significant shortcomings, primarily relying on traditional teaching methods in ward practice. This approach not only lacks opportunities for students to independently construct clinical reasoning frameworks but also fails to stimulate the cultivation of critical thinking and clinical decision-making abilities [4].

Driven by the rapid development of information technology, virtual simulation technology is deeply reconstructing the medical education ecosystem. Virtual standardized patients (VSPs), defined as artificial intelligence (AI)–driven, interactive virtual humans that simulate real patient encounters in 3D environments, offer a scalable alternative. As a product of the deep integration of computer multimedia technology and clinical medicine, VSPs have become an important carrier for revolutionizing clinical thinking training paradigms by accurately simulating real diagnosis and treatment scenarios through intelligent interactive systems [5-7]. Unlike static case studies, VSPs dynamically respond to learners’ actions, replicating nuanced clinical scenarios, from history-taking to diagnostic decision-making. With the rise of generative AI, modern VSP systems can now leverage natural language processing and machine learning to mimic diverse patient phenotypes, pathologies, and even emotional states, providing a near-lifelike training experience. In the field of international medical education, mature application systems like the DxR Clinician, developed by Southern Illinois University School of Medicine, have established benchmarks through AI-driven virtual patient simulations, dynamic clinical decision trees, and intelligent feedback mechanisms, making it a gold standard for clinical thinking training [8-10].

In contrast, VSP teaching in China is still in the exploratory stage of localization and adaptation and has not yet formed a large-scale application system. From the perspective of teaching implementation, the VSP system reconstructs the clinical competency development path through 3 core mechanisms [11]: first, constructing a full lifecycle disease spectrum database, covering typical and rare cases from newborns to the older population; second, creating a human-computer interactive consultation environment that requires learners to independently complete the entire process of medical history collection, physical examination operations, auxiliary examination selection, and treatment plan formulation; finally, relying on intelligent evaluation algorithms, millisecond-level feedback is provided to operational nodes, marking logical vulnerabilities and providing evidence-based medical treatment recommendations. This closed-loop system of “simulation practice, instant feedback, correction, and improvement” effectively breaks through the time and space limitations and ethical dilemmas of traditional bedside teaching.

Clinical empirical research shows that VSPs have unique advantages. In terms of diagnostic thinking, through the dynamic deduction of complex cases such as diabetic ketoacidosis, cultivate the ability to make timing decisions for differential diagnosis. In terms of treatment decision-making, individualized treatment plans based on drug metabolism characteristics are established through scenario simulations such as antibiotic tiered use [12]. In terms of humanistic literacy, a role-playing system for patients with depression is used to train their ability to analyze the social and psychological factors behind symptoms. This teaching paradigm of integrating reality and virtuality is reshaping the new landscape of clinical competence cultivation [13]. In recent years, the demand for medical safety in society has been constantly increasing, and the professional pressure on physicians has also increased accordingly. The long training period, heavy social responsibility, and high professional risks of physicians make it a key issue for medical education to improve skill levels in high-pressure environments. The virtual patient system provides a secure practice platform for medical students, allowing them to make mistakes in the virtual environment and learn from them, thereby reducing future medical errors in real clinical environments. This “trial and error learning” model not only helps cultivate high-level medical workers, but also provides new directions for the reform of medical education [14].

Traditional clinical internal medicine training heavily relies on case-based teaching, which often limits the development of students’ clinical reasoning skills and places substantial demands on instructors [15]. While VSPs offer a promising alternative, empirical evidence regarding their feasibility and effectiveness in enhancing clinical thinking remains limited. This study aimed to evaluate whether VSP-based training outperforms traditional methods in improving medical students’ clinical thinking abilities. Specifically, we addressed the following questions: (1) Does VSP training lead to greater improvements in theoretical knowledge and practical diagnostic skills compared to conventional case-based teaching? (2) Which components of clinical thinking are most influenced by VSPs? (3) How do learners perceive the utility of VSPs in their training? We hypothesized that the “VSPs in general practice” system, a 3D virtual platform simulating patient interactions, would significantly enhance clinical thinking metrics due to its immersive, repeatable scenarios. To test this, we conducted a randomized controlled trial (RCT) comparing VSP training with traditional teaching, assessing outcomes via standardized knowledge tests, virtual system scoring, and posttraining surveys. By rigorously evaluating VSPs, this study provides actionable insights into scalable, cost-effective alternatives to traditional clinical education, addressing gaps in evidence-based teaching innovations.

## Methods

### Trainee Recruitment

Based on the pilot data, we determined the sample size for this study to be 60 medical students. From a total pool of 85 eligible fifth-year undergraduate medical students interning at Quzhou People’s Hospital (affiliated with Wenzhou Medical University and Zhejiang University of Traditional Chinese Medicine), we recruited 60 participants via consecutive sampling between October 20, 2022, and October 20, 2024 (.

### Inclusion and Exclusion Criteria

We selected fifth-year undergraduate medical students from Wenzhou Medical University and Zhejiang University of Traditional Chinese Medicine who were interning at our hospital. The exclusion criteria were (1) participants who had received VSP or standardized patient training, (2) participated in courses related to clinical thinking skills, and (3) failure to comply with the research plan or withdrawal from the study.

### Randomization

Using computer-generated randomization, participants were randomly assigned to either the experimental group or the control group in a 1:1 ratio. Random grouping is conducted by individuals who have not had contact with the participants.

### Training Curriculum and Setting

The control group received traditional clinical thinking training through a combined problem-based learning and real-patient exposure approach. Supervisors selected patients from routine admissions in the Department of Internal Medicine who met the criteria for typical clinical cases outlined in the teaching syllabus, prioritizing broad alignment with the VSP curriculum while preserving real-world clinical heterogeneity. No further screening for specific attributes was applied to maintain ecological validity. Students were divided into small groups (10 per group) to: (1) conduct standardized clinical workflows (history-taking, physical examinations, and interpretation of laboratory and auxiliary tests); (2) formulate preliminary diagnoses and treatment plans; (3) participate in structured problem-based learning discussions based on these real medical records. Supervisors provided systematic theoretical explanations and feedback throughout the process. All patient interactions adhered to hospital protocols, with explicit consent obtained for student involvement. While exact case matching with the VSP group was logistically unfeasible, supervisors ensured thematic consistency to mitigate exposure variability between groups.

**Figure 1.**
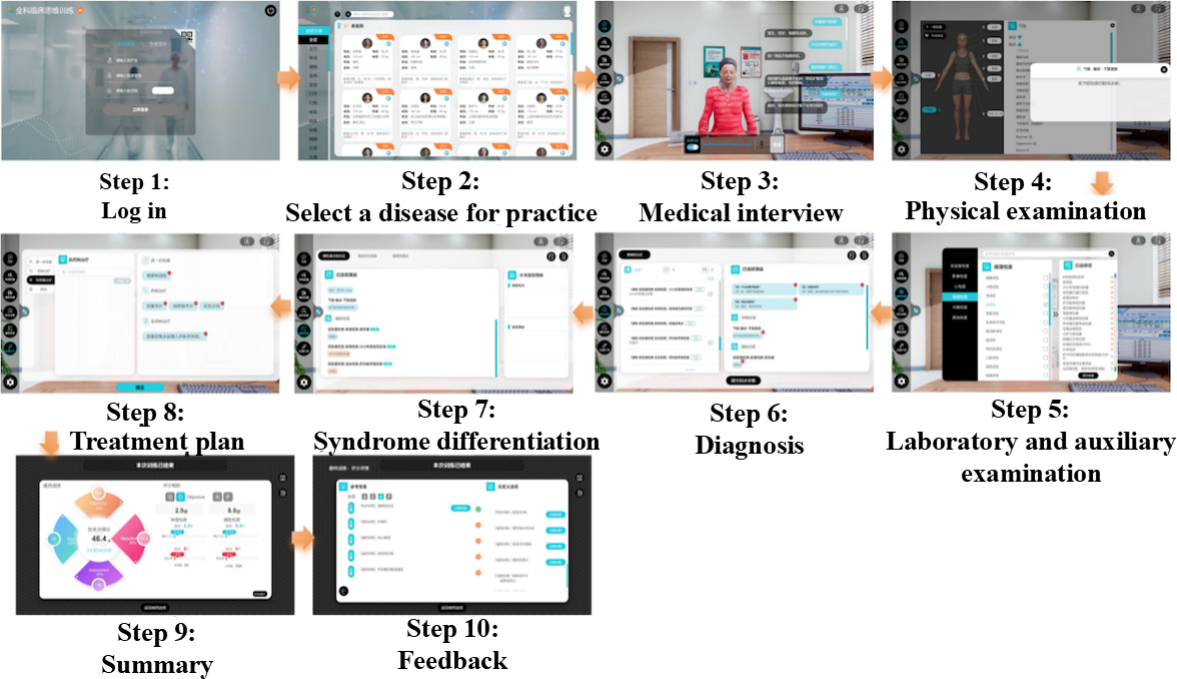
Operation procedures of the virtual standardized patient system.

The experimental group used the VSPs of the “VSPs in general practice” interactive diagnosis and treatment teaching system for teaching. The study used the HIWILL VSP System (version 2.1; Huawei Medical Technology), an interactive 3D clinical simulation platform designed for clinical thinking training. Each student in the experimental group completed 12 standardized VSP cases during the training period, with each case representing a different common clinical scenario in internal medicine. Students were allowed up to 3 attempts per case to achieve optimal performance, with immediate feedback provided after each attempt. The operation process of the VSP teaching system is shown in Figure 1.

### Selection and Improvement of VSPs

By using the VSPs included in the “VSPs in general practice” interactive diagnosis and treatment teaching system, the chief complaints, disease descriptions, consultations and physical examinations, laboratory and auxiliary examinations, as well as clinical cases required to be mastered in the treatment and teaching syllabus of VSPs are organically combined.

### Application of Virtualization Teaching Software

Implement VSP teaching for experimental group students, and teachers provide guidance and training on software usage methods for students. Students form small groups of 10 and complete the entire clinical diagnosis and treatment process through interaction with VSPs. Obtain the patient’s condition description, chief complaint, and abnormal signs in a virtual clinical environment. Based on the obtained consultation information, perform examinations on VSPs, including physical examination, laboratory examination, and, if necessary, histopathological examination. Students conducted a preliminary diagnosis of VSPs, provided corresponding diagnostic criteria, and then treated the disease. Teachers provide guidance and systematic explanations and training on relevant professional theoretical knowledge. All teachers supervising VSP sessions completed a standardized 8-hour training program that included: technical operation of the VSP system, standardized facilitation techniques, methods for providing consistent feedback, and protocols for troubleshooting technical issues. Instructors were required to pass a competency assessment before supervising sessions (Multimedia Appendix 1).

### Evaluation of Training Effectiveness

All students underwent medical record analysis exams and assessments of relevant professional theoretical knowledge upon enrollment. A 50-item multiple-choice exam covering core internal medicine topics was developed by a panel of 5 senior clinicians. And their clinical thinking abilities were evaluated through the intelligent module of the “VSPs in general practice” interactive diagnosis and treatment teaching system. The system evaluated performance across 4 domains: consultation skills (history taking and communication), diagnostic accuracy (initial and differential diagnoses), treatment planning (evidence-based interventions), and overall clinical reasoning (logical progression and efficiency). The score was recorded as the base score *F*_0_, and they entered group teaching. After completing the teaching tasks, all students underwent medical record analysis exams and assessments of relevant professional theoretical knowledge again, and their clinical thinking abilities were reevaluated through the intelligent module of the “VSPs in general practice” interactive diagnosis and treatment teaching system. The score was recorded as *F*_1_-score. Traditional case-based evaluations were structured to mirror the VSP domains but used paper-based scenarios. Both groups were assessed by blinded instructors using identical criteria.

The VSP system test primarily encompassed 4 components, with the medical interview section accounting for 25 points. This section evaluated the student’s ability to gather relevant medical history through human-computer dialogue. The condition examination section was worth 30 points, comprising a physical examination worth 15 points and auxiliary examinations worth 15 points. It assessed the student’s capability to perform necessary physical examinations based on the collected information and to select relevant tests and laboratory investigations required for diagnosis and differential, including histopathological examinations when necessary. The diagnosis and differential section carried 25 points, consisting of an initial diagnosis worth 16 points and a differential diagnosis worth 9 points. This section tested the student’s ability to choose the correct diagnostic criteria based on known information to make an accurate diagnosis and to analyze inclusion and exclusion criteria to complete the differential diagnosis. The treatment plan section was worth 20 points, with medication management accounting for 17 points and nonpharmacological interventions for 3 points. It evaluated the student’s competence in providing the correct treatment plan for the condition.

### Reference Standard for VSP Scoring

The VSP scoring algorithm was cross-validated against blinded expert evaluations (3 senior clinicians) who reviewed 20% of randomly selected VSP sessions. Interrater reliability (Fleiss κ=0.81) confirmed consistency between automated and manual scoring. Discrepancies were resolved by consensus.

### Feedback Questionnaire

Based on the characteristics of this study and reference literature, a self-designed learning effect questionnaire was used to investigate the effectiveness of prehospital emergency training among students. It mainly includes 5 questions, namely (1) beneficial for improving the ability to collect medical history, (2) it is conducive to improving the ability to analyze the condition, (3) beneficial for improving diagnostic and differential diagnostic capabilities, (4) beneficial for improving clinical treatment capabilities, and (5) it is conducive to improving clinical thinking ability. The evaluation criteria are divided into 4 levels: full agreement, agreement, partial agreement, and disagreement, which are filled in by students based on their true feelings. Those who fully agree or agree in the questionnaire will be considered as positive reviews, and the overall positive review rate and individual positive review rate will be comprehensively calculated. In addition, we conducted a questionnaire survey on 6 teachers responsible for clinical training to evaluate the potential impact of VSP courses on their work.

### Statistical Analysis

SPSS 26.0 statistical software (IBM Corp) was used to perform statistical processing on the data obtained from this study, analyzing the differences in scores between each group of students before and after teaching, and analyzing the differences in *F*_0_ and *F*_1_-scores between the 2 groups of students, in order to evaluate the differences in clinical thinking ability between students before and after teaching and under 2 different teaching modes. Continuous variables are represented as mean (SD), while categorical variables are represented as frequency or percentage. All continuous outcomes were analyzed using independent *t* tests, reporting mean differences, 95% CIs, and Cohen *d* as effect size measures. For categorical outcomes, Chi-square tests with odds ratios (ORs) and 95% CIs were used.

### Ethical Considerations

This study, which involved medical students as participants in an educational intervention evaluation, was granted a formal exemption from requiring full ethical review by the Quzhou People’s Hospital Medical Ethics Review Committee (review number: 2025‐004). This exemption is in full compliance with the institution’s policy, as well as the institutional guidelines for educational research. Specifically, the study was deemed to fall under the category of low-risk educational interventions that are part of the normal evaluation of curriculum quality and teaching methods. The anonymity of the participants was ensured, and the data were collected and analyzed in an aggregated manner for research purposes only, posing minimal risk to the participants. All participants provided written informed consent before participating in the study and received corresponding remuneration according to hospital regulations after the study ended.

## Results

### The Basic Characteristics of Participants

**Figure 2.**
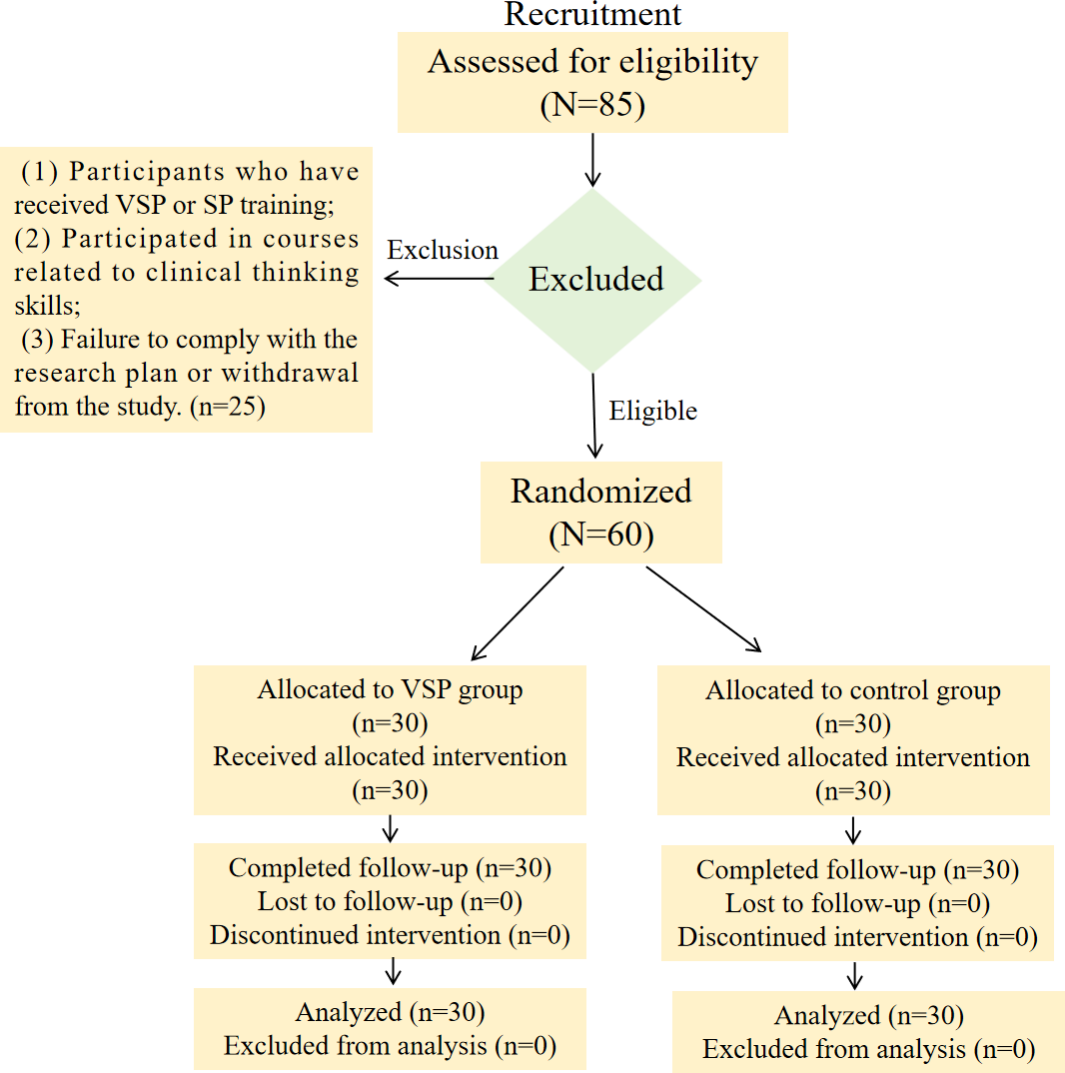
CONSORT (Consolidated Standards of Reporting Trials) flow diagram. SP: standardized patient training; VSP: virtual standardized patient.

There were 30 students in the control group, including 18 females and 12 males, with a mean age of 21.73 (SD 0.85) years. The experimental group consisted of 30 students, including 16 females and 14 males (mean age 21.60, SD 0.73) years (Figure 2). The prehospital emergency training for both groups is 6 months, and the training teachers for both groups are the same 6 teachers (2 bishops and 4 assistants). The average credit scores of the 2 groups of students were 3.66 (SD 0.36) points and 3.58 (SD 0.32) points, respectively. There were no statistically significant differences between the control group and the experimental group in terms of gender, age, average credit scores, training time, and training teachers, as shown in Table 1.

**Table 1.** General information of 2 groups of students.

	Control group (n=30)	Experimental group (n=30)	*t* test (*df*)	*P* value
Sex, n (%)			0.620 (59)	.28
Male	12 (40.0)	14 (46.7)		
Female	18 (60.0)	16 (53.3)		
Age (years), mean (SD)	21.73 (0.85)	21.60 (0.73)	3.454 (59)	.18
Grade point, mean (SD)	3.66 (0.36)	3.58 (0.32)	0.046 (59)	.75

### Evaluation of Training Effectiveness

#### Systematic Knowledge Test

**Figure 3.**
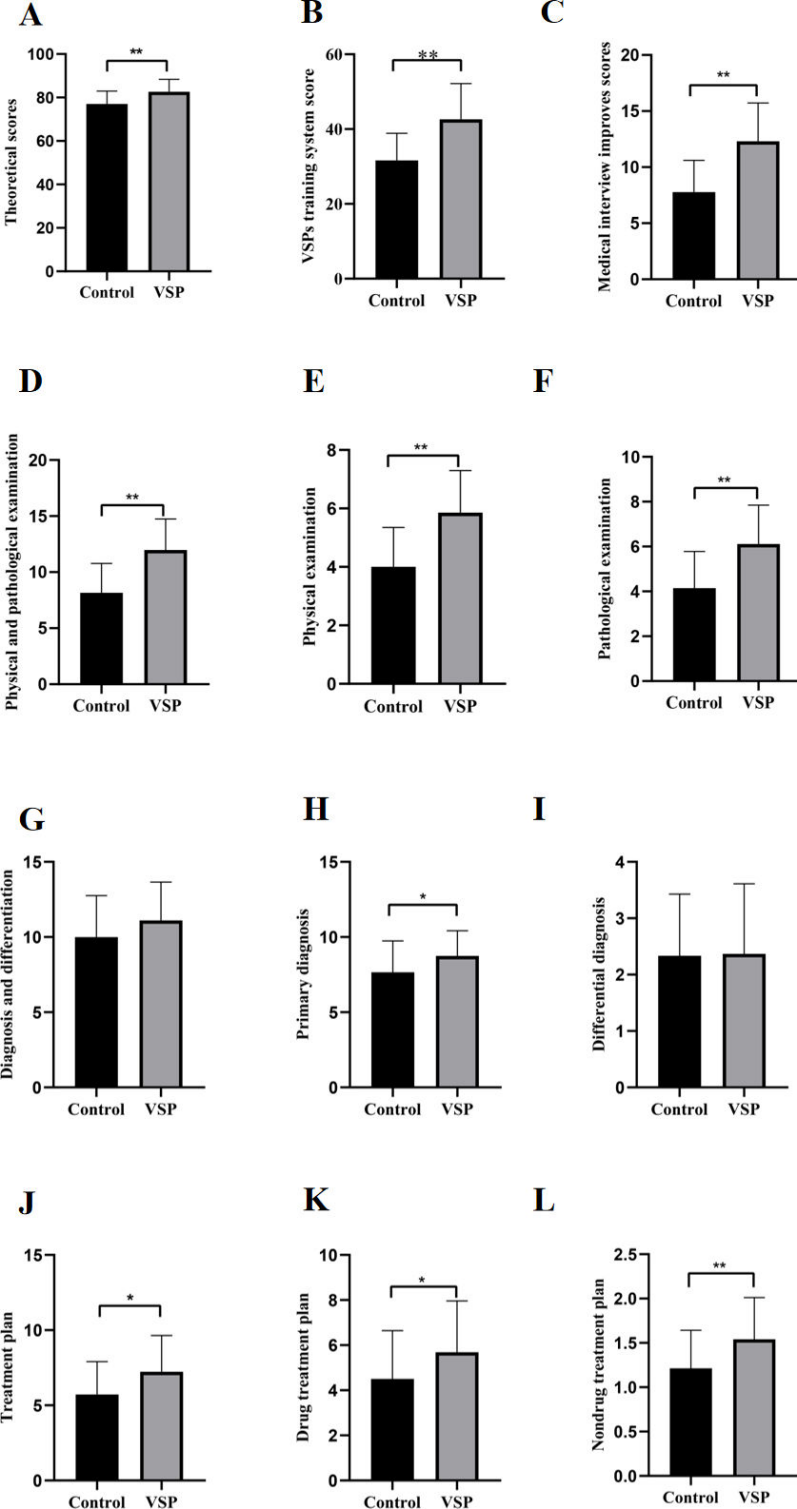
Results of formative evaluation, including (A) theoretical score, (B) VSP training system improvement score, (C) medical interview improvement score, (D) score improvement for physical examination and auxiliary examination, (E) physical examination improvement score, (F) pathological examination improvement score, (G) improvement of identification and diagnostic abilities, (H) improvement of primary diagnosis ability, (I) improvement of differential diagnosis ability, (J) improvement of treatment plan ability, (K) enhancement of drug treatment plan ability, and (L) enhancement of nondrug treatment plan ability. VSP: virtual standardized patient. *P<.05, **P<.01.

Figure 3A displays the theoretical performance results of 2 groups of trainees before and after training. Prior to clinical training, there was no significant difference in the theoretical performance between the experimental group and the control group (mean 65.83, SD 7.11 vs mean 66.57, SD 7.17; *F*_1, 59_=0.16; Cohen *d*=0.10; 95% CI 64.36-68.03; *P*=.69). After the training, the theoretical performance of the experimental group was significantly better than that of the control group (mean 82.63, SD 5.76 vs mean 77.07, SD 5.89; *F*_1, 59_=13.71; Cohen *d*=0.87; 95% CI 78.19-81.51; *P*<.001). Additionally, the improvement in theoretical scores was significantly greater in the experimental group compared to the control group (mean 17.07, SD 4.24 vs mean 10.67, SD 4.91; *F*_1, 59_=29.20; Cohen *d*=1.15; 95% CI 12.43-15.31; *P*<.001).

#### Clinical Skill Test

The test results of the virtual clinical reasoning training system are shown in Figure 3B. Before the clinical training, there was no significant difference in the total scores of the virtual clinical reasoning training system test between the experimental group and the control group (mean 35.90, SD 8 vs mean 35.70, SD 9.55; *F*_1, 59_=0; Cohen *d*=0.01; 95% CI 33.5-38; *P*=.96). After 4 weeks of training, the total scores of the virtual clinical reasoning training system test in the experimental group were significantly higher than those in the control group (mean 78.30, SD 7.98 vs mean 67.43, SD 8.74; *F*_1, 59_=25.27; Cohen *d*=1.09; 95% CI 70.30-75.44; *P*＜.001). Additionally, the improvement in the total scores of the virtual clinical reasoning training system test was significantly greater in the experimental group compared to the control group (mean 42.60, SD 9.56 vs mean 31.63, SD 7.24; *F*_1, 59_=25.10; Cohen *d*=1.09; 95% CI 34.51-39.72; *P*＜.001).

### Medical Interview

Figure 3C illustrates the scores of medical history-taking abilities for both groups of medical students before and after the training. After 4 weeks of training, the medical history-taking ability scores of the medical students in the experimental group (mean 20, SD 2.33 vs mean 15.45, SD 5.70; *F_1, 59_*=49.61; Cohen *d*=1.35; 95% CI 16.86-18.60; *P*<.001) and the improvement in medical history-taking ability scores (mean 12.30, SD 3.43 vs mean 7.77, SD 2.84; *F*_1, 59_=31.09; Cohen *d*=1.17, 95% CI 9.04-11.04; *P*<.001) were significantly higher than those in the control group.

### Physical Examination and Auxiliary Examination

Figure 3D-F displays the scores of the ability to conduct patient examinations for both groups of medical students before and after the training. After 4 weeks of training, the medical students in the experimental group showed significantly greater improvement in physical examination ability (mean 5.86, SD 1.44 vs mean 4, SD 1.34; *F*_1, 59_=26.41; Cohen *d*=1.11; 95% CI 4.50-5.36; *P*<.001) and in auxiliary examination ability (mean 6.11, SD 1.74 vs mean 4.15, SD 1.63; *F*_1, 59_=20.36; Cohen *d*=1.01; 95% CI 4.63-5.63; *P*<.001) compared to the control group. Additionally, the total improvement in objective examination scores was significantly higher in the experimental group than in the control group (mean 11.97, SD 2.77 vs mean 8.15, SD 2.62; *F*_1, 59_=30.08; Cohen *d*=1.16; 95% CI 9.21-10.91; *P*<.001).

### Diagnosis and Differentiation

Figure 3 depicts the scores of the ability to diagnose and perform differential diagnoses for both groups of medical students before and after the training. After 4 weeks of training, the medical students in the experimental group showed a significantly greater improvement in initial diagnosis scores (mean 8.74, SD 1.67 vs mean 7.66, SD 2.08; *F*_1, 59_=4.21; Cohen *d*=0.55; 95% CI 7.70-8.70; *P*=.045) compared to the control group. However, there was no significant difference between the 2 groups in the improvement of differential diagnosis ability (mean 2.37, SD 1.24 vs mean 2.33, SD 1.09; *F*_1, 59_=0.01; Cohen *d*=0.03; 95% CI 2.05-2.65; *P*=.91).

### Treatment Plan

Figure 3J-L displays the scores of the ability to administer treatment to patients for both groups of medical students before and after the training. After 4 weeks of training, the medical students in the experimental group showed significantly greater improvement in medication management ability (mean 5.69, SD 2.27 vs mean 4.51, SD 2.14; *F*_1, 59_=4.29; Cohen *d*=0.62; 95% CI 4.51-5.69; *P*=.04) and in nonpharmacological treatment ability (mean 1.54, SD 0.47 vs mean 1.21, SD 0.43; *F*_1, 59_=7.85; Cohen *d*=0.68; 95% CI 1.25-1.50; *P*=.007) compared to the control group. Additionally, the improvement in the overall ability to provide treatment to patients was significantly higher in the experimental group than in the control group (mean 7.23, SD 2.41 vs mean 5.72, SD 2.19; *F*_1, 59_=6.42; Cohen *d*=0.63; 95% CI 5.85-7.01; *P*=.01). The medium-large effect size (*d*=0.75) suggests that VSP training is not only statistically significant but also practically important in improving clinical reasoning. However, the wide CI (0.55-1.17) for Cohen *d* suggests that there is uncertainty about the true effect size and further validation is needed.

### Questionnaire

After the training, 2 groups of clinical medicine students who participated in the training were invited to conduct a satisfaction survey. As shown in Table 2, 29 of 30 medical students (96.7%) in the experimental group believe that VSP training is beneficial for improving their ability to collect medical history and analyze medical conditions. The experimental group of 27 of 30 medical students (90%) believes that VSP training is beneficial for improving diagnostic and differential diagnostic abilities, clinical treatment abilities, and clinical thinking abilities.

**Table 2.** Results of the learning effectiveness questionnaire.

Questionnaire content	Control group, n	VSP^a^ group, n	Chi-square (*df*)	*P* value
Beneficial for improving the ability to collect medical history			31.86 (59)	<.001
Strongly agree	5	22		
Agree	11	7		
Neutral	12	1		
Disagree	2	0		
Beneficial for improving the ability to analyze the condition			23.17 (59)	<.001
Strongly agree	12	27		
Agree	8	2		
Neutral	6	1		
Disagree	4	0		
Beneficial for improving diagnostic and differential diagnostic capabilities			22.35 (59)	＜.001
Strongly agree	7	23		
Agree	13	4		
Neutral	8	2		
Disagree	2	1		
Beneficial for improving clinical treatment capabilities			20.19 (59)	=.009
Strongly agree	10	19		
Agree	7	8		
Neutral	10	2		
Disagree	3	1		
Beneficial for improving clinical thinking ability			20.56 (59)	＜.001
Strongly agree	4	20		
Agree	11	7		
Neutral	9	2		
Disagree	6	1		

a VSP: virtual standardized patient.

## Discussion

### Principal Findings

This study found that VSPs can consolidate the basic knowledge of internal medicine among medical students, improve their clinical thinking ability, and enhance their ability to work independently. Clinical reasoning ability is one of the essential core competencies for medical students, requiring not only a solid theoretical foundation but also continuous accumulation through clinical practice by interacting with patients. Unlike clinical skills, which can be improved through repeated training on models, the VSPs in the “VSPs in general practice” interactive diagnostic and therapeutic teaching system are characterized by immersion, interactivity, and conceptualization [16]. The system establishes a simulated human model based on real clinical cases, incorporating various multimedia elements, such as images, sounds, and text to realistically recreate clinical environments. It allows real-time interaction through natural methods like vision and touch, simulating the clinical diagnosis and treatment processes. The system enables virtual examinations and treatments, including medical interviews, electrocardiogram examinations, physical examinations, laboratory tests, imaging studies, clinical interventions, medication administration, and device-based interventions [17]. All examinations performed by trainees on the virtual patients automatically provide feedback on the results. The VSPs present the patient’s condition and physical signs through lifelike 3D imaging, creating an immersive experience. Through an open diagnostic and therapeutic model, the system fully cultivates trainees’ ability to identify and solve problems, fostering clear, rigorous, and efficient thinking patterns. A comprehensive assessment system objectively evaluates the trainees’ clinical reasoning logic and mastery of basic skills. Under the premise of ensuring medical safety, it provides trainees with practice opportunities that closely resemble real clinical scenarios [15,18].

With the advancement of technology, VSPs have gradually become an important tool in medical education. VSPs are computer-based and AI-driven simulation tools designed to cultivate clinical reasoning abilities in medical students. By simulating real clinical cases and providing an interactive learning environment, VSPs help medical students improve their diagnostic, therapeutic, and communication skills [7,19,20]. This study suggests that, compared to traditional clinical training, VSP-based training may enhance medical students’ consolidation of theoretical knowledge and improve their abilities in clinical history-taking, physical and auxiliary examinations, diagnosis and differential diagnosis, as well as treatment planning. The findings indicate that virtual patient systems could contribute to reforming medical education curricula and support the goal of cultivating clinically competent professionals. VSPs offer several potential advantages: they can be reused indefinitely, allowing students to practice repeatedly in various scenarios without being influenced by the emotions or physical conditions of real patients, thus providing a safe practice environment. Additionally, VSPs may offer personalized feedback and guidance based on students’ learning progress and performance. While the initial development costs are high, VSPs could, in the long term, reduce the overall costs of medical education.

This RCT compared the teaching effectiveness of virtual training and traditional academic training for more than 4 weeks. The results suggest that students experienced less stress in the simulated clinic environment, which may have allowed them to dedicate more time to learning. Through repeated practice in a safe setting, they appeared to become more familiar with diagnostic and therapeutic processes. By the midterm assessment, students in the VSP group showed improvement in comprehensive abilities such as medical interviewing and clinical judgment compared to the control group. However, further research is needed to validate these findings across diverse educational settings and larger cohorts. Summative evaluation results also indicated that the VSP simulation system might enhance students’ theoretical knowledge, medical interview skills, syndrome differentiation, and treatment capabilities. Compared to traditional academic training, the use of standardized patient teaching appeared to improve students’ interpersonal communication skills, potentially helping them establish more harmonious doctor-patient relationships in future practice. While these findings are promising, additional studies are required to assess the long-term impact of VSP training on clinical performance and patient outcomes. Despite some technical and methodological challenges, ongoing advancements in virtual simulation technology are expected to further refine VSPs, potentially making them a valuable tool in medical education. However, their full integration into curricula will depend on continued validation and adaptation to different learning environments.

### Limitations

#### Content and Design Limitations

First, the VSP system primarily focuses on textbook-style disease presentations, potentially neglecting case variations and atypical scenarios encountered in real clinical practice. Additionally, the predefined decision-tree branches may restrict students’ exploration of alternative diagnostic hypotheses, limiting the simulation’s flexibility. Future research should enhance VSPs’ realism and adaptability to better mimic clinical complexity.

#### Methodological Constraints

This study relied on self-reported data and lacked objective external evaluation, which may introduce instructor-dependent variability. While the sample size was adequate for detecting primary outcome differences, it limited subgroup analyses. Furthermore, the control group’s nonstandardized patient pool, though intentionally designed to reflect real-world clinical training environments, could lead to confounding due to uneven case exposure. Multicenter studies with larger, standardized samples are needed to validate these findings.

#### Assessment and Long-Term Validity

The VSP-embedded assessment provided real-time metrics, but its alignment with the intervention might introduce measurement bias. To address this, we cross-validated results with standardized exams and faculty evaluations; however, future studies should incorporate third-party assessment tools. Moreover, while the study demonstrated significant short-term improvements in clinical knowledge and skills, the lack of long-term follow-up and direct validation with real patient interactions leaves the clinical relevance of these gains uncertain. Structured evaluations in real-world settings are warranted to confirm the predictive validity of VSP training.

### Conclusions

This RCT demonstrates the feasibility of VSPs in improving the clinical competence of medical students. VSPs deserve more attention and promotion. Therefore, future research aims to improve the VSPs system to more effectively enhance the clinical thinking ability of medical students. Future studies should use a hybrid evaluation approach, where trainees first complete VSP training and are then assessed with standardized real patient encounters, to establish the translational validity of virtual training to clinical practice.

## Supplementary material

10.2196/73196 Multimedia Appendix 1 Visual abstract.

10.2196/73196 Checklist 1 CONSORT checklist.

## Abbreviations

AI: artificial intelligence
CONSORT: Consolidated Standards of Reporting Trials
RCT: randomized controlled trial
VSP: virtual standardized patient
