# Supplementary figures and images for: Virtual Standardized Patients for Improving Clinical Thinking Ability Training in Residents: Randomized Controlled Trial
Source: JMIR Med Educ. 2025 Dec 8;11:e73196. doi: 10.2196/73196 (PMC12685284; doi:10.2196/73196)

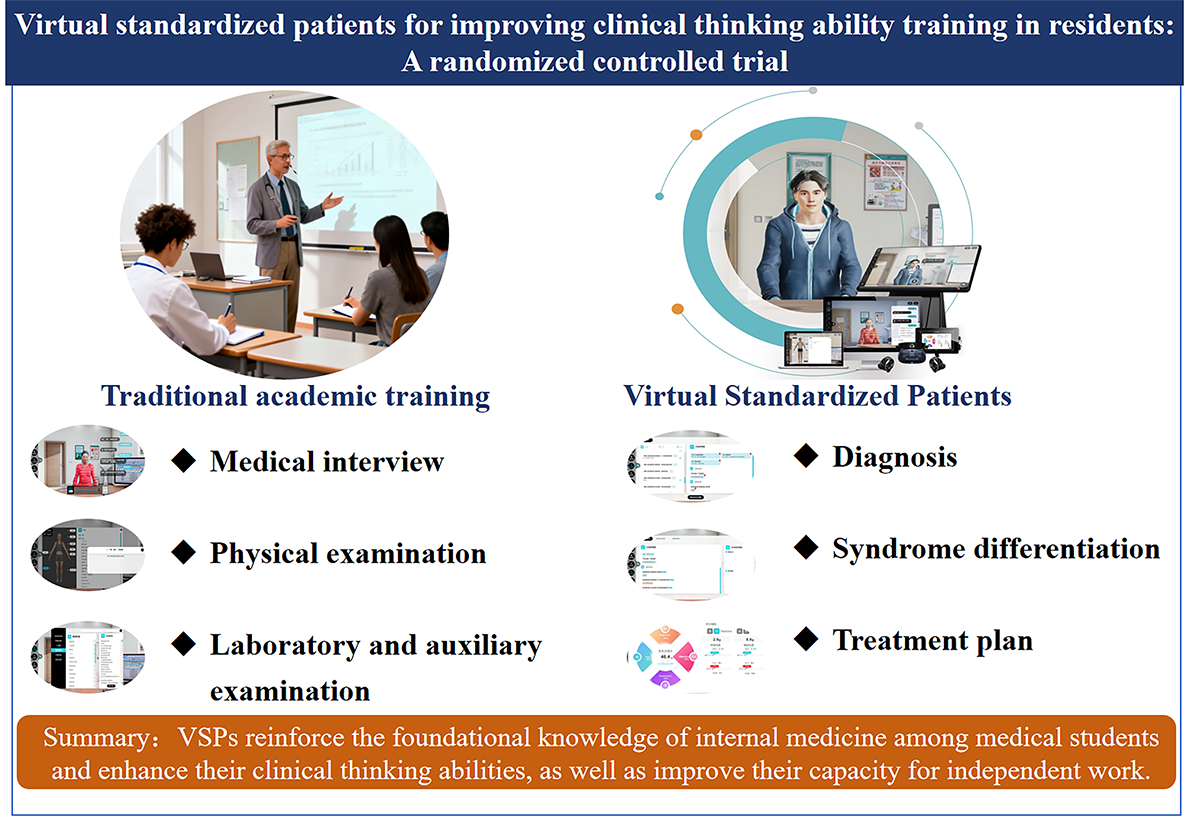

Supplement: Multimedia Appendix 1 [file mededu-v11-e73196-s001.png]
