# Supplementary material for: Virtual Standardized Patients for Improving Clinical Thinking Ability Training in Residents: Randomized Controlled Trial
Source: JMIR Med Educ. 2025 Dec 8;11:e73196. doi: 10.2196/73196 (PMC12685284; doi:10.2196/73196)
Supplement: Checklist 1 [file mededu-v11-e73196-s002.docx]

### ****CONSORT Checklist of Information to Include When Reporting a Randomized Trial****

| **Section/Topic** | **Item No.** | **CONSORT Checklist Item** | **Reported on Page No./Location in Your Manuscript** |
| --- | --- | --- | --- |
| **TITLE and ABSTRACT** |  |  |  |
|  | 1a | Identification as a randomized trial in the title. | **Title**: "Virtual standardized patients for improving clinical thinking ability training in residents: A randomized controlled trial" |
|  | 1b | Structured summary of trial design, methods, results, and conclusions. | **Abstract** (clearly structured under: Background, Objective, Methods, Results, Conclusions) |
| **INTRODUCTION** |  |  |  |
| Background and objectives | 2a | Scientific background and explanation of rationale. | **Introduction** (Paragraphs 1-5) |
|  | 2b | Specific objectives or hypotheses. | **Introduction** (Final paragraph: "Specifically, we addressed the following questions... We hypothesized that...") |
| **METHODS** |  |  |  |
| Trial design | 3a | Description of trial design (e.g., parallel, factorial) including allocation ratio. | **Methods: Randomization** ("...randomly assigned... in a 1:1 ratio.") - Implies a two-arm, parallel design. |
|  | 3b | Any changes to trial methods after trial commencement (e.g., eligibility criteria), with reasons. | No changes were made after trial commencement. |
| Participants | 4a | Eligibility criteria for participants. | **Methods: Inclusion and exclusion criteria** |
|  | 4b | Settings and locations where the data were collected. | **Methods: Trainee recruitment** ("...interning at Quzhou People’s Hospital...") |
| Interventions | 5 | The interventions for each group with sufficient details to allow replication. | **Methods: Training curriculum and setting** (Detailed descriptions for both Control and Experimental groups) |
| Outcomes | 6a | Completely defined pre-specified primary and secondary outcome measures, including how and when they were assessed. | **Methods: Evaluation of training effectiveness** (Defines theoretical exam and VSP system scores across 4 domains, assessed at enrollment (F0) and post-training (F1)). |
|  | 6b | Any changes to trial outcomes after the trial commenced, with reasons. | No changes to the trial outcomes were made after commencement. |
| Sample size | 7a | How sample size was determined. | **Methods: Trainee recruitment** ("Based on the pilot data, we determined the sample size... to be 60") |
|  | 7b | When applicable, explanation of any interim analyses and stopping guidelines. | Not applicable. (N/A) |
| Randomization: |  |  |  |
| Sequence generation | 8a | Method used to generate the random allocation sequence. | **Methods: Randomization** ("Using computer-generated randomization...") |
| Allocation concealment mechanism | 8b | Mechanism used to implement the allocation sequence, describing any steps taken to conceal the sequence until interventions were assigned. | **Methods: Randomization** ("Random grouping is conducted by individuals who have not had contact with the participants.") - This implies allocation concealment. |
| Implementation | 9 | Who generated the allocation sequence, who enrolled participants, and who assigned participants to interventions. | **Methods: Randomization** (Implies computer generated sequence, independent individuals assigned participants). |
| Blinding | 10a | If done, who was blinded after assignment to interventions (e.g., participants, care providers, those assessing outcomes) and how. | **Methods: Evaluation of training effectiveness** ("Both groups were assessed by blinded instructors..."). (Participants and teachers were likely not blinded due to the nature of the intervention). |
|  | 10b | If relevant, description of the similarity of interventions. | **Methods: Training curriculum and setting** (Describes efforts for thematic consistency between groups, same teachers, similar group sizes). |
| Statistical methods | 11a | Statistical methods used to compare groups for primary and secondary outcomes. | **Methods: Statistical analysis** |
|  | 11b | Methods for additional analyses, such as subgroup analyses and adjusted analyses. | No additional analyses were performed. |
| **RESULTS** |  |  |  |
| Participant flow (a diagram is strongly recommended) | 13a | For each group, the numbers of participants who were randomly assigned, received intended treatment, and were analyzed for the primary outcome. | **Results: The basic characteristics** and **Abstract** (n=60 randomized, n=30 per group, all completed). |
|  | 13b | For each group, losses and exclusions after randomization, together with reasons. | **Abstract** ("All enrolled medical students completed the study.") and **Results** imply no losses. |
| Recruitment | 14a | Dates defining the periods of recruitment and follow-up. | **Methods: Trainee recruitment** ("...between October 20, 2022 and October 20, 2024.") |
|  | 14b | Why the trial ended or was stopped. | Ended after reaching target sample size. |
| Baseline data | 15 | A table showing baseline demographic and clinical characteristics for each group. | **Results: The basic characteristics of participants** and **Table 1** |
| Numbers analyzed | 16 | For each group, number of participants (denominator) included in each analysis and whether the analysis was by original assigned groups. | **Results** (All analyses appear to include all 30 participants per group, consistent with Intention-to-Treat (ITT) as there were no dropouts). |
| Outcomes and estimation | 17a | For each primary and secondary outcome, a summary of results for each group, and the estimated effect size and its precision (e.g., 95% confidence interval). | **Results: Evaluation of training effectiveness** and **Figure 2** (Reports means, SDs, P-values, Cohen's d, and 95% CIs for score improvements). |
|  | 17b | For binary outcomes, presentation of both absolute and relative effect sizes is recommended. | **Results: Questionnaire** and **Table 2** (Reports counts and percentages; could be enhanced with Odds Ratios (ORs) as mentioned in the statistical plan). |
| Ancillary analyses | 18 | Results of any other analyses performed, including subgroup analyses and adjusted analyses, distinguishing pre-specified from exploratory. | Not mentioned. (N/A) |
| Harms | 19 | All important harms or unintended effects in each group. | During this study, no adverse events or unexpected effects of intervention measures were collected or reported. |
| **DISCUSSION** |  |  |  |
| Limitations | 20 | Trial limitations, addressing sources of potential bias, imprecision, and, if relevant, multiplicity of analyses. | **Discussion: Limitations** (A comprehensive paragraph is provided) |
| Generalizability | 21 | Generalizability (external validity, applicability) of the trial findings. | **Discussion** (Addressed in the 'Limitations' section and in the conclusion regarding future multi-center studies). |
| Interpretation | 22 | Interpretation consistent with results, balancing benefits and harms, and considering other relevant evidence. | **Discussion** (The entire section interprets results in the context of existing literature). |
| **OTHER INFORMATION** |  |  |  |
| Registration | 23 | Registration number and name of trial registry. | This experiment is not registered as it belongs to the research of educational curriculum evaluation. |
| Protocol | 24 | Where the full trial protocol can be accessed, if available. | The full study protocol is available from the corresponding author upon reasonable request. |
| Funding | 25 | Sources of funding and other support, role of funders. | **Acknowledgements** ("This study was funded by the Teaching Reform Project JG2022153 of Wenzhou Medical University." ) |
